# The Goat Cytotoxic T Lymphocyte-Associated Antigen-4 Gene: mRNA Expression and Association Analysis of Insertion/Deletion Variants with the Risk of Brucellosis

**DOI:** 10.3390/ijms252010948

**Published:** 2024-10-11

**Authors:** Congliang Wang, Xiaoyu Liu, Zhaofei Ren, Xiaomin Du, Na Li, Xiaoyue Song, Weiwei Wu, Lei Qu, Haijing Zhu, Jinlian Hua

**Affiliations:** 1 Shaanxi Centre of Stem Cells Engineering & Technology, College of Veterinary Medicine, Northwest Agriculture & Forestry University, Xianyang 712100, China; wcl1996@nwafu.edu.cn (C.W.); lina2017@nwsuaf.edu.cn (N.L.); 2 Shaanxi Provincial Engineering and Technology Research Center of Cashmere Goats, Life Science Research Center, Yulin University, Yulin 719000, China; lxy000825@163.com (X.L.); 15891221869@163.com (Z.R.); songxiaoyue@yulinu.edu.cn (X.S.); ylqulei@126.com (L.Q.); 3 Key Laboratory of Livestock Biology, Northwest Agriculture & Forestry University, Xianyang 712100, China; duxiaomin@nwafu.edu.cn; 4 Institute of Animal Science, Xinjiang Academy of Animal Husbandry Sciences, Urumqi 830000, China; wuweiweigp@foxmail.com

**Keywords:** *CTLA4*, InDel, goat, *Brucella*, inflammation

## Abstract

The cytotoxic T lymphocyte-associated antigen-4 (*CTLA4*) gene, a member of the immunoglobulin superfamily, is crucial for maintaining immune homeostasis and preventing autoimmune diseases. Studies have shown that polymorphisms in the *CTLA4* gene are linked to an increased risk of brucellosis in humans, but its association with brucellosis in goats remains unexplored. In this study, the tissue expression profile of *CTLA4* in goats was investigated, and the correlation between InDel polymorphisms in the *CTLA4* gene and susceptibility to brucellosis in goats was examined. The findings reveal the widespread expression of *CTLA4* in goat tissues, particularly in the spleen and testes. The tested goat populations presented genotypes insertion/insertion (II), insertion/deletion (ID), and deletion/deletion (DD) at both the P1 and P2 loci, and an association analysis revealed significant differences in the distribution of genotypes and allele frequencies at the P1 and P2 loci of the *CTLA4* gene between the *Brucella* goat case and the control groups (*p* < 0.05). Specifically, compared with the II genotype, the P1 and P2 loci were significantly associated with an elevated risk of brucellosis development in goats under both the codominant (ID/II) and dominant (ID + DD/II) models (P1, *p* = 0.042, *p* = 0.016; P2, *p* = 0.011, *p* = 0.014). Additionally, haplotype analysis indicated that haplotypes I_P1_D_P2_, D_P1_I_P2_, and D_P1_D_P2_ were significantly associated with an increased risk of brucellosis in goats compared to the reference haplotype I_P1_I_P2_ (*p* = 0.029, *p* = 0.012, *p* = 0.034). Importantly, the Lipopolysaccharide (LPS) stimulation of peripheral blood monocytes and/or macrophages from goats with the II, ID, and DD genotypes resulted in increased *CTLA4* expression levels in the II genotype, leading to a robust LPS-induced inflammatory response. Through bioinformatic analysis, the observed effect of the InDel locus on *Brucella* pathogenesis risk in goats could be attributed to the differential binding of the transcription factors nuclear factor kappaB (NF-κB) and CCAAT/enhancer-binding protein α (C/EBPα). These findings offer potential insights for breeding strategies against brucellosis.

## 1. Introduction

*Brucella* is a Gram-negative and intracellular facultative coccidia that can infect more than 60 mammalian species, including humans [1], sheep [2], and goats [3]. Ruminants such as cattle and sheep are the primary hosts, with infection leading to late-term fetal loss, stillbirth, reduced milk production, reproductive disorders in females, and inflammatory reactions such as enlarged testes or testicular inflammation in males [4]. Among the known *Brucella* species, *B. melitensis* is recognized as the most virulent and infectious, and it can be transmitted through various routes, including the digestive tract, mucous membranes, and broken skin [5]. The period during which livestock give birth poses a significant risk for *Brucella* outbreaks. Additionally, high-density farming environments and interregional livestock transportation contribute to the accelerated spread of *Brucella* and subsequent infections. Humans can become infected through multiple routes, such as the consumption of raw meat and milk, as well as through contact with aborted animals [6]. The pathogenesis of *Brucella* in livestock and humans is notably similar; when *Brucella* enters the host, it rapidly traverses the mucosal epithelium to reach the lymph nodes and other immune organs. While the majority of these bacteria are phagocytosed and eliminated by macrophages, a small number of survivors persist, and these survivors interact with the endoplasmic reticulum to form replicative *Brucella* vacuoles (rBCVs), where they undergo extensive replication, subsequently infecting other tissues and cells as they circulate in the bloodstream [7]. The Shaanbei White Cashmere goat, a distinctive breed in western China known for its high-quality cashmere and meat production, exhibits strong adaptability and significant reproductive potential, contributing to its economic value [8]. However, the main obstacle to efficient reproduction in these goats is *B. melitensis* infection, which can lead to female infertility, miscarriage, or male orchitis [4]. While developed countries have successfully controlled livestock brucellosis, it continues to be a concern in developing regions such as Asia, the Middle East, and Latin America [9].

In China, a major livestock-farming nation, the prevalence of total brucellosis in sheep and goats has been increasing in recent years, with a national average of 2.3%; in certain provinces, the prevalence rate can reach 18.7%, and the national prevalence of brucellosis in dairy cattle stands at 1.9% [10,11]. In Tanzania, the overall national herd prevalence of brucellosis is 8.2% [12], with some herds in the northern rangelands of Kenya experiencing rates as high as 15% [13]. A high prevalence of 14.14% in livestock has been reported in the Middle East, where *Brucella* is endemic [14]. Brucellosis, caused by *Brucella* infections, is a significant global zoonotic disease that poses a serious threat to public health and results in substantial economic losses in livestock production [1]. The economic losses attributed to *Brucella* infection per goat have been documented to reach USD 30.80 in India and USD 162.55 in Malaysia [15,16]. Furthermore, the incidence of human cases of *Brucella* infection exceeds 500,000 globally each year [17], with over 90% of these cases stemming from *B. melitensis* [18]. Slaughter and vaccination are crucial methods for preventing and controlling the spread of brucellosis in cattle, sheep, and other populations. However, challenges have arisen, such as the difficulty in differentiating between naturally infected animals and those that have been immunized via common serological diagnostic tests like RBPT, STAT, and ELISA [19]. Additionally, vaccination may not always be effective and could lead to abortions in pregnant animals, complicating the elimination and purification of brucellosis. Although some live attenuated brucellosis vaccines, such as the *Brucella* M5-90Δbp26 mutant, have been developed to differentiate between vaccine immunity and natural infection [20], they require the use of the ELISA technology, increasing the costs and economic burdens on farms.

Host genetic factors have been shown to be associated with either resistance or susceptibility to *Brucella* [21]. Immune responses differ among individuals due to various genetic polymorphisms in immune-related genes, including insertions/deletions (InDels) and single-nucleotide polymorphisms (SNPs), which play a role in regulating inflammatory signaling pathway activities. Screening genetic markers for brucellosis resistance can be an effective strategy to combat brucellosis and minimize economic losses. Marker-assisted selection (MAS) has become a common method for identifying genetic markers related to traits such as litter size, growth, and disease resistance in goats. Single-nucleotide polymorphisms (SNPs) and insertions/deletions (InDels) are the most commonly used markers [22], allowing for accurate analyses of individual livestock genetic composition for genotypic selection. These measures can increase the efficiency of livestock breeding. Research indicates that *CTLA4*, a member of the immunoglobulin superfamily, plays a crucial role in negatively regulating T-cell proliferation and activation [23]. In immune homeostasis, *CTLA4* functions by blocking the binding of T-cell receptors to costimulatory molecules through its interaction with B7, and this action ultimately reduces T-cell activation and expansion, thus maintaining immune tolerance [24]. Furthermore, *CTLA4* also decreases the antigen-presenting capacity of dendritic cells by inhibiting the expression of costimulatory molecules [25]. Studies have indicated that overexpression or functional defects in *CTLA4* can disrupt immune tolerance and increase the risk of autoimmune diseases, including systemic lupus erythematosus [26].

Inflammatory factors such as IFN-γ and TNF-α, which derive from the Th1 immune response, play crucial roles in resistance to *Brucella* infection and proliferation [27]. However, *Brucella* infection can undermine the protective immune response, resulting in the increased circulation of regulatory T-cells (Tregs) within the patient’s body, and the expression of *CTLA4*, a significant immune checkpoint for Tregs, is markedly elevated in individuals with chronic brucellosis, suggesting that *Brucella* may induce the overexpression of immune checkpoints in Tregs, potentially leading to the impaired control of *Brucella* infection [28]. More importantly, the T allele and CT genotype of the −318 C/T variant locus in the *CTLA4* gene have been linked to an increased risk of brucellosis infection in humans [29]. Given previous research connecting *CTLA4* gene variations with immune and inflammatory diseases, we hypothesized that variations in the goat *CTLA4* gene could also be associated with brucellosis infections, but few reports on this have been published. Therefore, this study aimed to identify variant loci in the *CTLA4* gene that may be linked to brucellosis risk in goats, offering valuable insights for breeding strategies to control brucellosis in these animals.

## 2. Results

### 2.1. Gene Conservation Analysis and mRNA Expression

**Figure 1:**
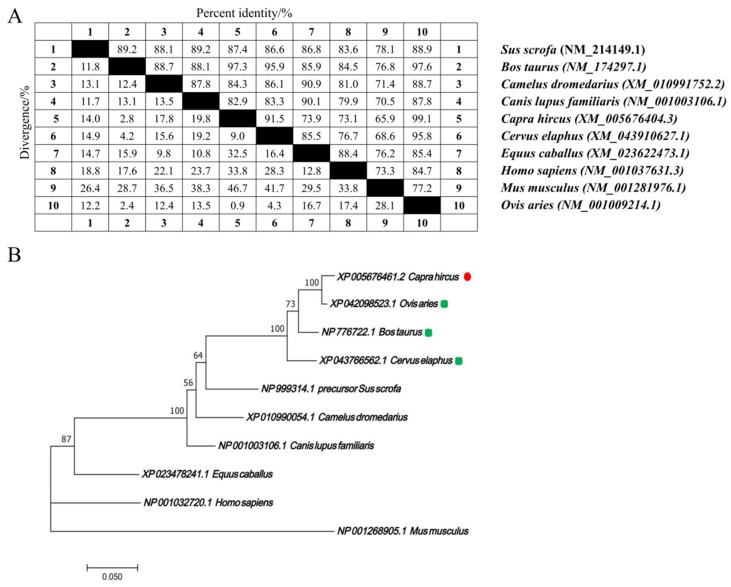
*CTLA4* gene bioinformatic analysis in goats: (**A**) nucleotide sequence homology analysis of *CTLA4* gene; and (**B**) phylogenetic tree of *CTLA4* gene in different animal species.

**Figure 2:**
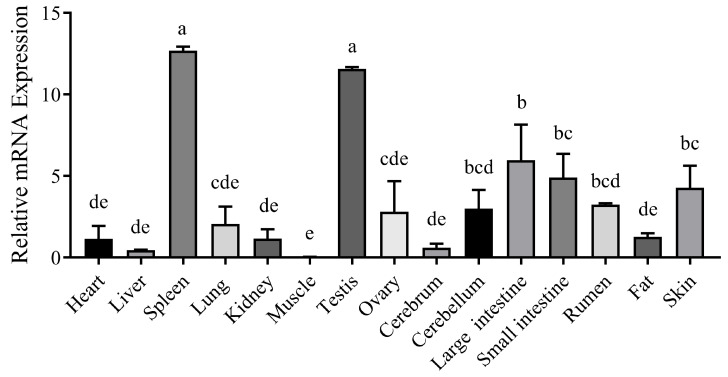
Tissue expression profile of *CTLA4* gene. *n* = 3 samples of each tissues. Columns with different letters (a–e) mean *p* < 0.05.

The nucleotide sequences of the *CTLA4* gene from 10 different species were retrieved from GenBank and analyzed via the MegAlign software (version 7.1.0). The results indicated that the goat *CTLA4* gene shared high homology with *Ovis aries* (99.1%), *Bos taurus* (97.3%), and *Cervus elaphus* (91.5%). Conversely, it showed lower homology with *Mus musculus* (65.9%) and *Homo sapiens* (73.1%) (Figure 1A). The phylogenetic tree constructed via the NJ joining method revealed that the genetic distance between the goat *CTLA4* gene and those of *Ovis* aries, *Bos taurus*, and *Cervus elaphus* was the closest, suggesting a close relationship with livestock species, particularly sheep and cattle, but a more distant relationship with *Homo sapiens* and *Mus musculus* (Figure 1B). These findings were consistent with the nucleotide sequence homology analysis. Furthermore, the qRT-PCR results demonstrated that *CTLA4* was broadly expressed in all the goat tissues, with the lowest expression in muscle and the highest in the spleen and testes (Figure 2), particularly the former, which is a vital immune organ.

### 2.2. Identification of Insertion/Deletion Variants in the CTLA4 Gene

**Figure 3:**
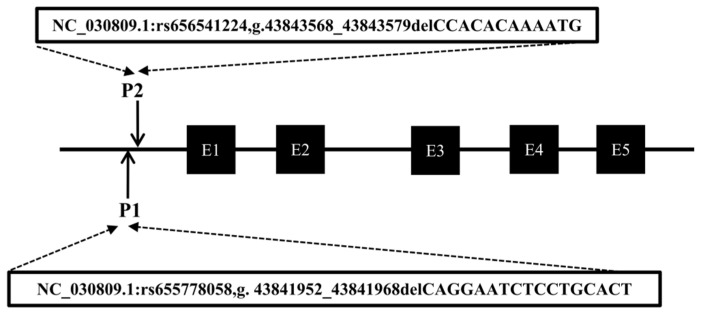
Mode pattern of identified indel positions of goat *CTLA4* gene. The black box represents the exons of the goat *CTLA4* gene.

**Figure 4:**
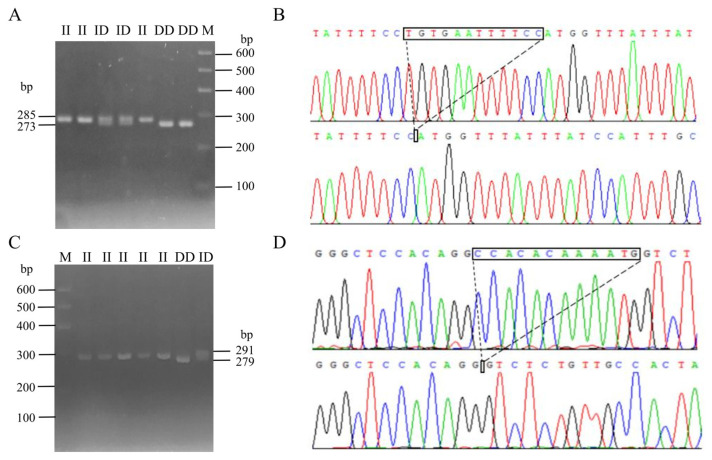
InDel electrophoresis and sequencing of *CTLA4* gene in goats: P1 locus electrophoresis (**A**) and sequencing map (**B**); and P2 locus electrophoresis (**C**) and sequencing map (**D**). M: 600 bp marker; II: insertion/insertion; ID: insertion/deletion; and DD: deletion/deletion.

Following the method described in a previous study [30], DNA pool samples were subjected to PCR amplification to identify polymorphisms at the P1 and P2 loci of the *CTLA4* gene, and the locations of these two mutations are shown in Figure 3. Gel electrophoresis of agarose (Figure 4A,C) and sequencing analysis (Figure 4B,D) revealed polymorphic loci at both P1 and P2, each of which presented three genotypes: insertion/insertion (II), insertion/deletion (ID), and deletion/deletion (DD). The II genotype displayed a single band (287 bp and 291 bp), the ID genotype presented two bands (287 bp and 273 bp; 291 bp and 279 bp), and the DD genotype presented a single band (273 bp and 279 bp). Notably, the predicted mutation sequence at the P1 locus in the Ensembl database was ACTCAGGAATCTCCTGC/-, whereas the actual sequencing result was TGTGAATTTTCC/-. The mutation sequence at the P2 locus was aligned with the predicted sequence in the Ensembl database.

### 2.3. Analysis of Genetic Parameters of CTLA4 in Goats

Table 1 displays the sample size, genotype, and allele frequency of the *CTLA4* gene at the P1 and P2 loci in goats. A total of 804 and 838 goat genomes were successfully genotyped at the P1 and P2 variant sites, respectively. For both the P1 and P2 loci, the II genotype frequencies were greater across the case, control, and all goat populations (P1: 0.668, 0.586, and 0.627; P2: 0.728, 0.782, and 0.689), and the frequencies of the I alleles were consistently greater than those of the D alleles (P1: 0.804, 0.750, and 0.777; P2: 0.846, 0.806, and 0.826). The polymorphism information content (PIC) values revealed that the P1 locus presented moderate genetic diversity (0.25 < PIC < 0.50) across the case, control, and all goat populations. The genotype distributions at the P1 locus were all found to deviate from Hardy–Weinberg equilibrium (HWE) (*p* < 0.05). On the other hand, none of the genotype distributions at the P2 locus deviated from the HWE (*p* > 0.05). However, the P2 locus presented a low genetic diversity (0 < PIC < 0.25) across all goat populations.

### 2.4. Association Analysis of CTLA4 Gene Polymorphisms with the Risk of Brucellosis in Goats

Previous studies have indicated a link between the risk of human brucellosis infection and a specific SNP in the *CTLA4* gene [29]. As a key candidate gene potentially linked to brucellosis risk, we conducted a statistical analysis of the frequency distribution of genotypes and alleles at the P1 and P2 loci of the *CTLA4* gene within the goat population under study (Table 2). The results revealed significant differences in the distribution of the three genotypes (II, ID, DD) at the P1 and P2 loci between cases and controls (*p* < 0.05), as well as significant disparities in the I and D alleles between the two groups (*p* < 0.05). These findings suggest a potential association between certain genotypes or alleles at these loci and the risk of brucellosis.

Binary logistic regression analysis was used to investigate the associations between four genetic models (codominant, dominant, recessive, and allelic) and the risk of brucellosis at the P1 and P2 loci of the *CTLA4* gene (Table 3). The results indicated that, at the P1 locus, compared to the II genotype, the risk of brucellosis was 1.337 and 1.422 times greater in the codominant model (ID/II) and dominant model (ID + DD/II), respectively (*p* = 0.042, 95% CI = 0.968–1.846; and *p* = 0.016, 95% CI = 1.067–1.896). Additionally, the D allele conferred a 1.303-fold greater risk of brucellosis than the I allele (*p* = 0.009, 95% CI = 1.028–1.652). Similarly, at the P2 locus, relative to the II genotype, the risk of brucellosis was 1.512 and 1.448 times greater in the codominant model (ID/II) and dominant model (ID + DD/II), respectively (*p* = 0.011, 95% CI = 1.096–2.086; and *p* = 0.014, 95% CI = 1.079–1.944). Furthermore, the D allele was associated with a 1.326-fold greater risk of brucellosis than the I allele (*p* = 0.029, 95% CI = 1.029–1.710).

### 2.5. Linkage Disequilibrium (LD) and Haplotype Association Analysis

**Figure 5:**
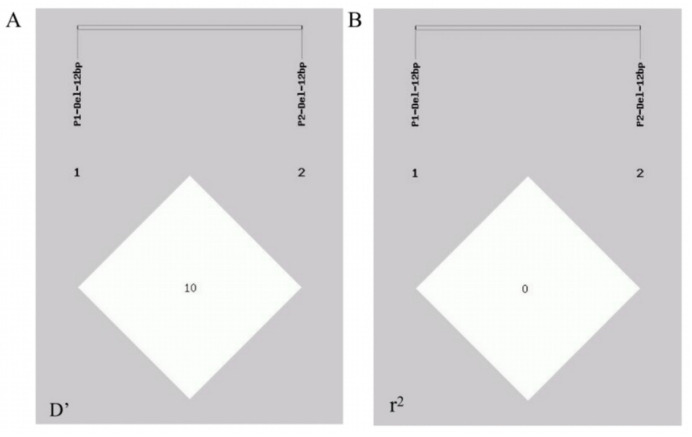
Linkage disequilibrium (LD) between P1 and P2 mutation locus of *CTLA4* gene in goats. (**A**) D’ value and (**B**) r^2^ value.

A linkage disequilibrium analysis was performed via the GDICALL website (http://www.msrcall.com/Gdicall.aspx; accessed on 12 July 2024) to explore the possibility of linkage between the two variant sites in the *CTLA4* gene (Figure 5), and the D’ and r^2^ values were 0.108 and 0.009, respectively, indicating that there was no strong link between the two variant loci.

Haplotype construction was performed on the two InDel loci of P1 and P2 (Table 4), and four haplotypes, I_P1_I_P2_, D_P1_I_P2_, I_P1_D_P2_, and D_P1_D_P2_, were generated. The results revealed that, when haplotype I_P1_I_P2_ was used as a reference, the risk of brucellosis increased by 1.416, 1.415, and 1.634 times with haplotypes D_P1_I_P2_, I_P1_D_P2_, and D_P1_D_P2_, respectively (*p* = 0.029, 95% CI = 1.037–1.933; *p* = 0.012, 95% CI = 1.078–1.858; and *p* = 0.034, 95% CI = 1.039–2.571).

### 2.6. LPS Stimulates Changes in CTLA4 and Cytokine mRNA Expression Levels in Peripheral Blood Monocytes and/or Macrophages

**Figure 6:**
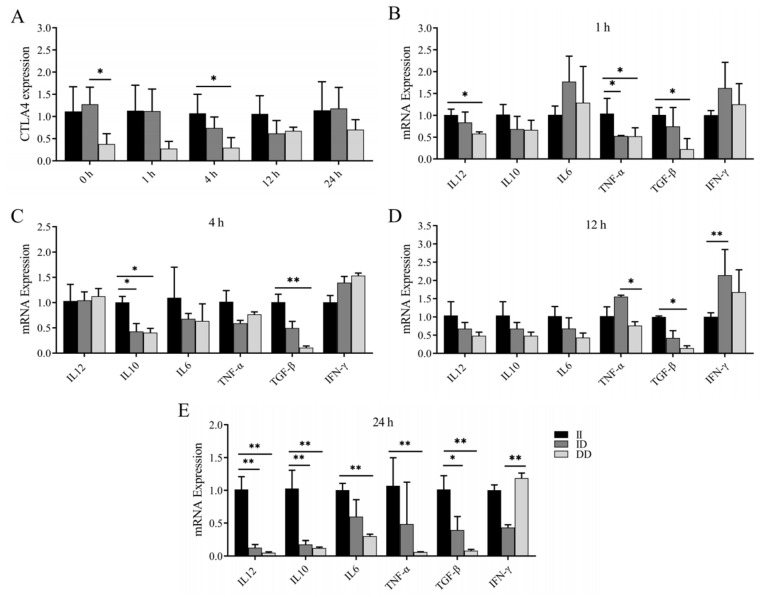
Changes in *CTLA4* and cytokines in peripheral blood monocytes and/or macrophages of goats of different genotypes after LPS stimulation. (**A**) Changes in *CTLA4* expression at different time points in LPS-stimulated peripheral blood monocytes and/or macrophages; and (**B**–**E**) changes in cytokine expression of *IL*-6, *IFN-γ*, *TNF-α*, *IL*-10, *IL*-12, and *TGF-β*, respectively, at different time points after LPS stimulation. * *p* < 0.05 and ** *p* < 0.01.

Previous studies have revealed an association between the P1 and P2 loci and susceptibility to brucellosis infection. In this study, we investigated the variations in *CTLA4* and cytokine mRNA expression levels in the peripheral blood monocytes and/or macrophages of goats with different genotypes following LPS stimulation. Our findings revealed that the expression of *CTLA4* in monocytes and/or macrophages of goats with the DD genotype was consistently lower than in those with the II genotype at various time points, with a significant difference observed after 4 h of LPS stimulation (*p* < 0.05) (Figure 6A). Additionally, at 1 h post LPS stimulation, the expression of *TNF-α* in goats with the II genotype was significantly greater than in goats with the ID and DD genotypes, whereas the expression of *IL*-12 and *TGF-β* was notably greater than in goats with the DD genotype (Figure 6B). Following 4 h of LPS stimulation, *IL*-10 expression was significantly elevated in goats with the II genotype compared with those with the ID and DD genotypes, and *TGF-β* expression was notably greater than in goats with the DD genotype (*p* < 0.05) (Figure 6C). Moreover, after 12 h of stimulation, *TGF-β* expression was significantly greater in goats with the II genotype than in those with the DD genotype, and *IFN-γ* expression was notably greater in goats with the ID genotype than in those with the II genotype (*p* < 0.05) (Figure 6D). Finally, following 24 h of stimulation, goats with the II genotype presented significantly higher expression levels of *IL*-12, *IL*-10, and *TGF-β* than those with the ID and DD genotypes, whereas the expression levels of *IL*-6 and *TNF-α* were notably greater than in goats with the DD genotype (*p* < 0.05) (Figure 6E).

### 2.7. Predicted Binding of Transcription Factors

**Figure 7:**
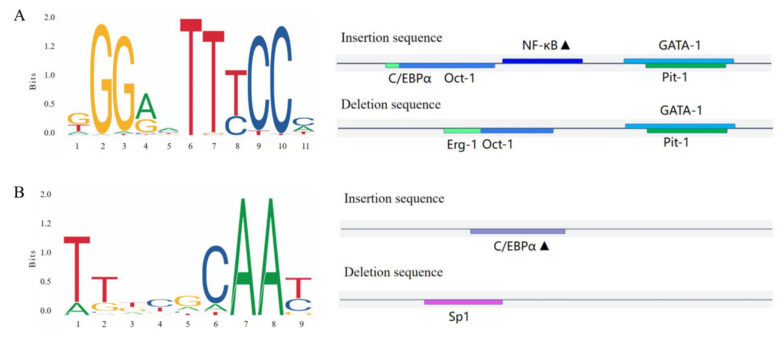
Transcription factor-binding site prediction for the goat *CTLA4* gene variant locus. (**A**,**B**) represent the P1 and P2 loci, and the black triangles represent potential transcription factor-binding sites.

Bioinformatics was utilized to predict the transcription factor-binding sites of the *CTLA4* gene variation sites in goats, as shown in Figure 7. The study revealed that the P1 (Figure 7A) and P2 (Figure 7B) insertion sequences of the *CTLA4* gene had the ability to specifically bind to nuclear factor kappaB (NF-κB) and the CCAAT/enhancer-binding protein α (C/EBP-α) transcription factors, respectively.

## 3. Discussion

Researchers widely recognize that *Brucella abortus* is associated with T lymphocyte apoptosis and is able to induce the production of Th1 proinflammatory cytokines, such as IFN-g and TNF-α, in infected hosts, which are involved in the regulation of macrophage activation and bacterial proliferation [31]. Moreover, the interaction between costimulatory and coinhibitory receptors on T-cells, such as *CTLA4*, with ligands on antigen-presenting cells (APCs), such as CD80 and CD86, can impact the strength and duration of antigen-specific T-cell responses [32]. Studies have shown that mice lacking *CTLA4* experience lymphocyte infiltration and tissue damage and ultimately develop lymphoproliferative disorders [33], highlighting the immunomodulatory role of *CTLA4* in activities such as lymphocyte infiltration and the regulation of T-cell proliferation and activation [34]. Given the important role of *CTLA4* in regulating T-cell activation and enhancing antigen-presenting capacity, researchers have combined the *CTLA4* immunoglobulin variable region (IgV_CTLA4) with novel multiepitope vaccine proteins to develop a new vaccine against brucellosis, and this vaccine demonstrates superior immunogenicity and antigenicity both in vitro and in vivo [35]. Additionally, *CTLA4* gene variants have been linked to basal cell carcinoma (BCC) [36], rectal cancer [37], acute kidney transplant rejection [38], and various inflammatory diseases [26]. While *CTLA4* gene variants have been linked to human brucellosis infection [29], research on their association with brucellosis in goats is lacking. Therefore, the relationship between *CTLA4* gene polymorphisms and brucellosis resistance in goats is worth exploring.

The sample sizes of existing and current studies on the relationship between genetic polymorphisms and brucellosis in goats are almost always small (*N* < 300) [39], which affects the accuracy and persuasiveness of the results to a certain extent; therefore, we investigated the relationship between *CTLA4* gene polymorphisms and brucellosis in a large sample size of Shaanbei White Cashmere goats (*N* > 800) and reported that two InDel loci of the *CTLA4* gene exhibited polymorphisms in these animals. Previous research has suggested that the potential mechanism behind this effect may be linked to a functional mutational polymorphism (LD) within the gene [40]; however, there is no LD between these two loci. In general, there is a direct relationship between the diversity in genetic variation and the evolutionary potential of a population, and a low genetic diversity results in the diminished adaptive capacity of individuals and the decreased reproductive capacity of the population [41]. Conversely, a higher genetic diversity enhances the evolutionary potential of the population and improves the overall immune response of the population when confronted with pathogen attacks [42]. Higher PIC values and population heterozygosity typically indicate greater genetic diversity [8], and, in this study, the PIC values and population heterozygosity of the P1 locus were found to be greater than those of the P2 locus. This finding suggests that the P2 locus has a lower level of genetic diversity, indicating that the intensity of artificial selection could be effectively intensified. In addition, the P2 locus conformed to HWE (*p* > 0.05), whereas the P1 locus deviated from HWE (*p* < 0.05), which may have been related to the fact that the tested population was subjected to high-intensity artificial selection and genetic drift, resulting in the loss of some alleles or the introduction of new mutations. In this study, when the II genotype was used as a reference, the risk of brucellosis was significantly greater in the codominant model (ID/II) and the dominant model (ID + DD/II) than in the II genotype (*p* < 0.05), the risk of brucellosis development was significantly greater for the D allele than for the I allele (*p* < 0.05), and the degree of risk of brucellosis was significantly greater for haplotypes D_P1_I_P2_, I_P1_D_P2_, and D_P1_D_P2_ than for haplotype I_P1_I_P2_ (*p* < 0.05). The above results suggest that the II genotype and I allele may be associated with resistance to brucellosis infection in goats; thus, individual II genotype goats may be intensively selected for brucellosis resistance for the total goat population. Notably, immunogenetic evolution in animals may be influenced by pathogen-mediated gene selection pressure, with ruminants being the primary hosts of *Brucella* infections [43]. Natural resistance-associated macrophage protein 1 (*Nramp1*) is recognized as a key gene associated with high levels of integrated disease resistance, and its genetic variation has been extensively documented in relation to *Brucella* resistance in livestock, including goats [43] and cattle [44]. Furthermore, the amino acid sequence of the goat *Nramp1* gene is more than 96% similar to those of cattle and sheep, indicating that *Brucella*-mediated host selection influences the evolutionary trajectory of this gene [45]. In our research, the nucleotide sequences of goat *CTLA4* presented the highest homology with those of sheep and cattle. The tree topology indicates that the *CTLA4* gene is highly conserved among livestock, including goats, sheep, and cattle, and suggests that the *CTLA4* gene may play a role in *Brucella* evolution.

Several studies have demonstrated correlations between gene polymorphisms, gene expression, and susceptibility to brucellosis. After *Brucella* infection in buffaloes, individuals with the BB genotype displayed a rapid increase in *Nramp1* expression in monocytes, along with a reduced number of intracellular bacteria [46]. The GA genotype with the rs7749323 variant of the *TNFAIP3* gene was linked to resistance against *Brucella* infection; however, the expression of the *TNFAIP3* gene in the monocytes of individuals with the GA genotype was notably lower than that of the GG genotype when exposed to lipopolysaccharide (LPS) and *Brucella* stimulation [47]. These results suggest a connection between the mRNA expression of genes and early resistance against pathogen invasion and warrant further investigation. The inflammatory response plays a crucial role in protecting the animal body against pathogen invasion, and immunological functions are carried out through monocyte and macrophage recognition of pathogenic bacteria, phagocytosis, and the secretion of inflammatory cytokines. Therefore, in this study, we stimulated monocytes and/or macrophages from goats with different genotypes via LPS. We observed that the expression of *CTLA4* in goat monocytes and/or macrophages of the DD genotype was significantly lower than that in the II genotype after 4 h of LPS stimulation. In particular, after 24 h of stimulation, the expression of IL-10, *IL*-12, and *TGF-β* in monocytes and/or macrophages of the II genotype was significantly greater than that in those of the ID and DD genotypes, and the expression of *IL*-6 and *TNF-α* was significantly greater than that in the DD genotype. Collectively, the evidence suggests that the II genotype or I allele acts as a protective factor against *Brucella* infection in goats. In a previous study assessing the immune response of *CTLA4* to the intracellular protozoan Trypanosoma cruzi, researchers reported a significant increase in *CTLA4* expression in the splenic T-cells of infected mice, and the blockade of *CTLA4* was shown to increase the production of *IFN-γ*, *TNF-α*, and NO, which significantly reduced both parasitemia and mortality in mice infected with the Y strain of *T. cruzi* and improved host resistance to the Y strain [48]. Similarly, elevated levels of *CTLA4* expression were observed in lymphocytes from patients with tuberculosis, and the blockade of *CTLA4* was found to enhance the immune response to *Mycobacterium tuberculosis* infection [49]. In our study, we noted that *CTLA4* expression levels in individual monocyte macrophages from genotype II goats increased after 4 h of LPS stimulation of the monocytes. Notably, within 24 h of LPS stimulation, *TNF-α* expression in individual monocyte macrophages from genotype II goats remained consistently high, thereby inducing a robust inflammatory response. Collectively, these findings indicate the involvement of *CTLA4* in the regulation of the LPS-mediated inflammatory response in monocytes and/or macrophages.

Previous studies have shown that mutations in noncoding regions of genes can impact gene binding to DNA sequences, transcription factors, and splicing factors, ultimately affecting gene expression and protein translation [50]. Therefore, we propose that the specific binding of transcription factors could be responsible for this effect. Using bioinformatics to predict transcription factor-binding sites, we determined that the insertion sequences of the two variants could bind to nuclear factor Kappab (NF-κB) and CCAAT/enhancer-binding protein α (C/EBPα). NF-κB signaling is closely linked to immunoregulatory processes such as lymphoid organ development, dendritic cell antigen presentation, and innate antiviral immunity [51] and plays a role in inflammatory regulation and immune maintenance in conditions such as rheumatoid arthritis, renal inflammation, and metabolic inflammation [52]. The activation of NF-κB is frequently associated with the upregulation of antiapoptotic gene expression, and this process induces the production of cytokines and adhesion molecules that regulate the immune response, including TNF-α, and facilitates the recruitment of leukocytes to sites of inflammation. Notably, bacterial and viral infections represent significant pathways for NF-κB activation [53]. On the other hand, C/EBPα is crucial for the TLR3-mediated production of inflammatory cytokines and immune response to viral infections, and the silencing or knocking down C/EBPα has been shown to result in more severe disease [54]. It has been reported that the transcription factor C/EBPα specifically binds to mC-6, a CpG site located in the promoter region of trefoil factor 1 (TFF1), and, by constructing a C/EBPα overexpression vector, co-transfecting it with the TFF1 promoter region into porcine intestinal epithelial cells infected with porcine epidemic diarrhea virus (PEDV), a significant reduction in the rate of PEDV infestation was observed, along with the inhibition of its replication [55]. Most variants associated with complex traits, such as disease resistance, are located in noncoding regions of the genome, which makes transcription factor–DNA binding interactions a crucial method for investigating gene expression, post-transcriptional regulation, and phenotypic changes [56]. In this study, the II genotype and the I allele were found to be linked to resistance against *Brucella* infection in goats, and the polymorphism in the *CTLA4* gene may affect gene expression and the immune response to pathogenic bacterial infection through differential binding of the transcription factors NF-κB and C/EBPα. However, it is essential to note that disease resistance is influenced by multiple genes, making it a complex trait that can be affected by various factors, including genetic background, regional environment, and individual developmental differences [57,58], and further comprehensive studies are needed to fully understand these interactions.

## 4. Materials and Methods

### 4.1. Samples and Data Collection

This study utilized 850 unrelated adult female Shaanbei White Cashmere goats from Shaanbei White Cashmere Goat Farm in Yulin city, Shaanxi province, China. Ear tissue samples were collected and stored in 75% ethanol for DNA extraction. Blood samples were obtained through jugular vein puncture and tested using the Rose Bengal Plate Agglutination Test (RBPT), as described by Rahman et al. [59]. Briefly, the RBPT involved mixing 20 μL of Rose Bengal plate antigen with goat serum on a slide, observing for 3~5 min, and comparing with positive and negative controls. The presence of agglutination indicated a positive result, while its absence indicated a negative result. Positive samples were re-examined for confirmation. All goats in this study were not vaccinated against brucellosis and were part of a high-incidence group (>30% seropositive for brucellosis), showing clinical symptoms like miscarriage and stillbirth. The goats were all raised in the same environment, resulting in an equal risk of *Brucella* infection. Tissue samples including heart, liver, spleen, lung, kidney, ovary, cerebrum, cerebellum, large intestine, small intestine, muscle, rumen, fat, and skin were collected from adult female goats, along with tissue samples from adult male goats (*n* = 3 per group) for gene expression analysis. All samples were immediately cryopreserved in liquid nitrogen for subsequent RNA extraction.

### 4.2. DNA Extraction, Primer Design, and Genotyping

Following the established protocol, DNA was extracted from ear tissue samples and assessed for purity and quality using a Nanodrop 2000 spectrometer (Thermo Scientific, Waltham, MA, USA) [60]. The extracted DNA was then diluted with RNA-free enzyme water to a concentration of 20 ng/μL and stored in a −20 °C refrigerator for genetic variation testing. A DNA pool was constructed using 48 randomly selected DNA samples from Shaanbei White Cashmere Goats to detect insertion/deletion mutations in the *CTLA4* gene. The reference sequence and InDel variation information of the goat *CTLA4* gene (GenBank accession NC_030809.1) were obtained from the NCBI (https://www.ncbi.nlm.nih.gov/; accessed on 25 April 2024) and Ensembl databases (http://asia.ensembl.org/index.html; accessed on 25 April 2024). Two pairs of primers were designed using the Primer Premier Software (version 5.0) to amplify specific fragments of the *CTLA4* gene (Table 5). PCR was carried out in a 13 μL reaction mixture, with steps including predenaturation at 95 °C for 5 min, followed by 40 cycles at 95 °C for 1 min, 59 °C for 20 s, and 72 °C for 20 s; the final step involved extension at 72 °C for 10 min and storage at 16 °C. Individual genotypes of the goats were determined through 3% agarose gel electrophoresis and a Fully Automated Gel Imaging System (Gel Doc EZ, BIO-RAD, Hercules, CA, USA). Genotypic sequencing was carried out by a company (Sangon Biotech, Shanghai, China).

### 4.3. RNA Extraction, cDNA Synthesis, and qRT-PCR

Total RNA was extracted from the tissue samples using TRIzol Total RNA Extraction Reagent (Takara, Dalian, China) following the manufacturer’s protocol. First-strand cDNA was synthesized with the Prime Script™ RT kit (Takara, Dalian, China) and stored at −80 °C. qRT-PCR primers were designed using the Primer Premier5.0 software (Table 5). qRT-PCR was conducted in a 20 mL system with a reaction mixture containing 2 × ChamQ SYBR qPCR Master Mix (Vazyme, Nanjing, China) 10 μL, cDNA template 1 μL, and 1 μL each of the upstream and downstream primers (10 pmol/μL for each primer), along with RNase-free ddH_2_O 8 μL. PCR reactions were conducted following a three-step reaction procedure [30], with GAPDH serving as the internal control gene. The relative expression of each tissue was assessed using the 2^−ΔΔCt^ method, with three repetitions for each tissue cDNA sample.

### 4.4. Bioinformatic Analysis

The nucleotide and protein sequences were downloaded from the NCBI database (https://www.ncbi.nlm.nih.gov/; accessed on 14 July 2024). A nucleotide sequence homology comparison analysis was conducted using MegAlign (version 7.1.0) (https://www.dnastar.com/; accessed on 14 July 2024) [61], the neighbor-joining method in MEGA (version 7.0.26) (http://www.megasoftware.net/; accessed on 14 July 2024) was utilized to construct the evolutionary tree [62], the bootstrap test was performed with a repetition rate of 1000-fold and a confidence interval of 95%, and other parameters were kept at their default values. The prediction of transcription factor-binding sites for the InDel site of the *CTLA4* gene was conducted through the AliBaba 2.1 website (http://gene-regulation.com/pub/programs/alibaba2/; accessed on 18 July 2024) and JASPAR (https://jaspar.genereg.net/; accessed on 18 July 2024).

### 4.5. LPS In Vitro Stimulation of Peripheral Blood Monocytes and/or Macrophages

Peripheral blood monocytes and/or macrophages were isolated from adult goats that tested negative in the Rose Bengal Plate Agglutination Test. The cells were obtained through density gradient centrifugation following the manufacturer’s instructions (TBD, Tianjin, China) and cultured in RM-1640 medium (Gibco, New York, NY, USA) supplemented with 10% fetal bovine serum (Gibco, New York, NY, USA), 1% non-essential amino acids (Gibco, New York, NY, USA), 1% β-Mercaptoethanol (Gibco, New York, NY, USA), and 1% dual antibodies (Solarbio, Beijing, China) for 48 h. Subsequently, the cells were stimulated with 1 μg/mL LPS (GENE-LAB, Hangzhou, China) for 0, 4, 12, and 24 h [63]. TRIzol total RNA extraction reagent was used to extract RNA from the monocytes and/or macrophages, and cDNA was synthesized to analyze the expression levels of cytokines such as *CTLA4*, *IL*-6, *IFN-γ*, *TNF-α*, *IL*-10, *IL*-12, and *TGF-β*. *GAPDH* was used as an internal control gene, the relative expression levels were quantified using the 2^−ΔΔCt^ method, and three replicates were performed for each cDNA sample.

### 4.6. Statistical Analysis

Population heterozygosity (He), homozygosity (Ho), and polymorphic information content (PIC), along with other genetic indicators, were calculated using the Nei method for the *CTLA4* gene variant locus [64]. Hardy–Weinberg equilibrium (HWE), linkage disequilibrium (LD) structures, and haplotype construction were assessed using the SHEsis platform (http://analysis.bio-x.cn; accessed on 12 July 2024) and the GDICALL website (http://www.msrcall.com/Gdicall.aspx; accessed on 12 July 2024), and the LD was considered strong if R^2^ > 0.33. The distribution frequencies of different genotypes and alleles were analyzed using the Chi-squared test in the SPSS 23.0 software. Additionally, a logistic regression model was utilized to calculate the odds ratio (OR) values and 95% confidence intervals (95% CIs) to evaluate the impact of various genetic models, alleles, or haplotypes on the risk of brucellosis [65].

## 5. Conclusions

This study reports the identification of two novel InDel mutations in the *CTLA4* gene within a population of Shaanbei White Cashmere Goats. Individuals possessing the II genotype or I allele of these InDels exhibited a reduced susceptibility to brucellosis. Stimulation of monocytes and/or macrophages with LPS revealed that those with the protective I allele demonstrated an increased secretion of anti-inflammatory cytokines, including *IL*-10, *IL*-12, and *TGF-β*. These findings underscore the role of the *CTLA4* gene in the immune response to brucellosis infections and may serve as a significant genetic marker for breeding goats with enhanced resistance to brucellosis.

## Figures and Tables

**Table 1:** Genetic parameters of polymorphic sites of the *CTLA4* gene.

Locus	Serological Results	Sizes	Genotypic Frequencies			Allelic Frequencies		Population Parameters				HWE
		N	II	ID	DD	I	D	Ho	He	Ne	PIC	*p* Value
P1	Cases	398	0.668 (266)	0.271 (108)	0.060 (24)	0.804	0.196	0.685	0.315	1.460	0.266	*p* < 0.05
	Control	406	0.586 (238)	0.328 (133)	0.086 (35)	0.750	0.250	0.625	0.375	1.600	0.305	*p* < 0.05
	Sum	804	0.627 (504)	0.300 (241)	0.073 (59)	0.777	0.223	0.653	0.347	1.531	0.287	*p* < 0.05
P2	Cases	416	0.728 (303)	0.236 (98)	0.036 (15)	0.846	0.154	0.740	0.260	1.352	0.227	*p* > 0.05
	Control	422	0.782 (274)	0.694 (132)	0.038 (16)	0.806	0.194	0.687	0.313	1.456	0.264	*p* > 0.05
	Sum	838	0.689 (577)	0.274 (230)	0.037 (31)	0.826	0.174	0.712	0.288	1.404	0.246	*p* > 0.05

**Table 2:** Genotypes and allele frequencies of polymorphic locus of *CTLA4* gene in *Brucella* cases and controls.

Locus	Genotype/Allele	Frequencies		χ^2^	*p* Value
		Cases	Control		
P1	II	0.668 (266)	0.586 (238)	6.121	0.047
	ID	0.271 (108)	0.328 (133)		
	DD	0.060 (24)	0.086 (35)		
	I	0.804 (640)	0.750 (609)	6.764	0.009
	D	0.196 (156)	0.250 (203)		
P2	II	0.728 (303)	0.782 (274)	6.473	0.039
	ID	0.236 (98)	0.694 (132)		
	DD	0.036 (15)	0.038 (16)		
	I	0.846 (704)	0.806 (680)	4.769	0.029
	D	0.154 (128)	0.194 (164)		

Note: *p* < 0.05, significant difference.

**Table 3:** Association between genetic modeling of polymorphic locus in the *CTLA4* gene and risk of brucellosis development.

Locus	Genetic Models	Genotype/Allele	Frequencies		*p* Value	OR (95% CI)
			Cases	Control		
P1	Codominant	II	0.668 (266)	0.586 (238)		1.00
		ID	0.271 (108)	0.328 (133)	0.042	1.337 (0.968~1.846)
		DD	0.060 (24)	0.086 (35)	0.081	1.501 (0.853~2.643)
	Dominant	II	0.668 (266)	0.586 (238)		1.00
		ID + DD	0.332 (132)	0.414 (168)	0.016	1.422 (1.067~1.896)
	Recessive	II + ID	0.940 (374)	0.914 (371)		1.00
		DD	0.060 (24)	0.086 (35)	0.161	1.470 (0.858~2.520)
	Alleles	I	0.804 (640)	0.750 (609)		1.00
		D	0.196 (156)	0.250 (203)	0.009	1.303 (1.028~1.652)
P2	Codominant	II	0.728 (303)	0.782 (274)		1.00
		ID	0.236 (98)	0.694 (132)	0.011	1.512 (1.096~2.086)
		DD	0.036 (15)	0.038 (16)	0.654	1.186 (0.556~2.532)
	Dominant	II	0.728 (303)	0.782 (274)		1.00
		ID + DD	0.272 (113)	0.218 (148)	0.014	1.448 (1.079~1.944)
	Recessive	II + ID	0.964 (401)	0.962 (406)		1.00
		DD	0.036 (15)	0.038 (16)	0.887	1.054 (0.514~2.160)
	Alleles	I	0.846 (704)	0.806 (680)		1.00
		D	0.154 (128)	0.194 (164)	0.029	1.326 (1.029~1.710)

Note: *p* < 0.05, significant difference. Abbreviations: CI, confidence interval; and OR, odds ratio.

**Table 4:** Relationship between *CTLA4* gene haplotypes and risk of brucellosis incidence.

Haplotypic Names	Haplotypic Types	Haplotypic Frequencies		*p* Value	OR (95% CI)
		Cases	Control		
Hap1	I_P1_I_P2_	529 (0.692)	476 (0.609)		1.00
Hap2	I_P1_D_P2_	84 (0.110)	107 (0.137)	0.029	1.416 (1.037–1.933)
Hap3	D_P1_I_P2_	117 (0.154)	149 (0.191)	0.012	1.415 (1.078–1.858)
Hap4	D_P1_D_P2_	34 (0.044)	50 (0.064)	0.034	1.634 (1.039–2.571)

Note: *p* < 0.05, significant difference. Abbreviations: CI, confidence interval; and OR, odds ratio.

**Table 5:** InDel detection and qRT-PCR primers for *CTLA4* gene in goats.

Primer	Primer Sequence (5′-3′)	Length (bp)	Tm (°C)	Function
P1	F: ATTCCATCACCCACAG R: ATTACCCAGTTATCCTTC	285	59	Indel detection
P2	F: CACAGCCATACCCATTC R: GCACCATAAGGAGCAA	291	59	Indel detection
*GAPDH*	F: AAAGTGGACATCGTCGCCAT R: CCGTTCTCTGCCTTGACTGT	116	60	Internal control
*CTLA4*	F: GGCAGCAGTTAGTTCAGGGT R: GCTCTGTTGGGGGCATTTTC	165	60	qPCR
*IL*-6	F: TTCAGTCCACTCGCTGTCTC R: TGCTTGGGGTGGTGTCATTC	106	60	qPCR
*IL*-10	F: TGATGCCACAGGCTGAGAAC R: TTCGCAGGGCAGAAAACGAT	119	60	qPCR
*IL*-12	F: AACCAGACCCACCCAAGAAC R: GTGGCATGTGACTTTGGCTG	197	60	qPCR
*IFN-γ*	F: AGATCCAGCGCAAAGCCATA R: TCTCCGGCCTCGAAAGAGAT	110	60	qPCR
*TNF-α*	F: AACCCATCTACCAGGGAGGG R: AGTAGACCTGCCCAGACTCA	108	60	qPCR
*TGF-β*	F: AACAATTCCTGGCGCTACCT R: ACTGAGGCGAAAGCCCTCTA	135	60	qPCR

## Data Availability

The data/models supporting the findings of this study are available from the authors upon request.
